# A small gene sequencing panel realises a high diagnostic rate in patients with congenital nystagmus following basic phenotyping

**DOI:** 10.1038/s41598-019-49368-7

**Published:** 2019-09-13

**Authors:** Luke O’Gorman, Chelsea S. Norman, Luke Michaels, Tutte Newall, Andrew H. Crosby, Christopher Mattocks, Angela J. Cree, Andrew J. Lotery, Emma L. Baple, J. Arjuna Ratnayaka, Diana Baralle, Helena Lee, Daniel Osborne, Fatima Shawkat, Jane Gibson, Sarah Ennis, Jay E. Self

**Affiliations:** 1 Human Development and Health, Faculty of Medicine, University of Southampton, MP808, Tremona Road, Southampton, SO16 6YD UK; 2 Clinical and Experimental Sciences, Faculty of Medicine, University of Southampton, MP806, Tremona Road, Southampton, SO16 6YD UK; 3 Eye Unit, University Hospital Southampton NHS Foundation Trust, Tremona Road, Southampton, SO16 6YD UK; 4 Institute of Biomedical and Clinical Science, University of Exeter Medical School, RILD Wellcome Wolfson Centre, Exeter, EX2 5DW UK; 5 Wessex Investigational Science Hub, University Hospital Southampton, Tremona Road, Southampton, SO16 6YD UK; 6 Medical Research (Level 4), University of Exeter Medical School, RILD Wellcome Wolfson Centre, Royal Devon and Exeter NHS Foundation Trust, Barrack Road, Exeter, EX2 5DW UK; 7 Biological Sciences, Faculty of Natural and Environmental Sciences, University of Southampton, Southampton, SO17 1BJ UK; 8 Human Genetics & Genomic Medicine, Faculty of Medicine, University of Southampton, MP 808, Tremona Road, Southampton, SO16 6YD UK

**Keywords:** Data processing, Disease genetics, Molecular medicine

## Abstract

Nystagmus is a disorder of uncontrolled eye movement and can occur as an isolated trait (idiopathic INS, IINS) or as part of multisystem disorders such as albinism, significant visual disorders or neurological disease. Eighty-one unrelated patients with nystagmus underwent routine ocular phenotyping using commonly available phenotyping methods and were grouped into four sub-cohorts according to the level of phenotyping information gained and their findings. DNA was extracted and sequenced using a broad utility next generation sequencing (NGS) gene panel. A clinical subpanel of genes for nystagmus/albinism was utilised and likely causal variants were prioritised according to methods currently employed by clinical diagnostic laboratories. We determine the likely underlying genetic cause for 43.2% of participants with similar yields regardless of prior phenotyping. This study demonstrates that a diagnostic workflow combining basic ocular phenotyping and a clinically available targeted NGS panel, can provide a high diagnostic yield for patients with infantile nystagmus, enabling access to disease specific management at a young age and reducing the need for multiple costly, often invasive tests. By describing diagnostic yield for groups of patients with incomplete phenotyping data, it also permits the subsequent design of ‘real-world’ diagnostic workflows and illustrates the changing role of genetic testing in modern diagnostic workflows for heterogeneous ophthalmic disorders.

## Introduction

Infantile nystagmus syndrome (INS) is a condition which can be present as an isolated trait (idiopathic INS, IINS) or as part of a plethora of ocular or systemic disorders including albinism, retinal disease and neurological disorders. IINS is most commonly seen either in singletons, or in X-linked pedigrees. Tarpey *et al*.^1^ reported that 57% of putative X-linked pedigrees and 94% of proven X-linked pedigrees harbour causal mutations in the FERM domain containing 7 gene, *FRMD7*^1^. Although *FRMD7* mutations are the only known genetic cause of IINS, many disorders are known to masquerade as IINS in children. These conditions are often missed due to the difficulty in identifying associated phenotypes in children (such as hypomorphic albinism^2^) or a delay in the onset of additional clinical features (such as spino-cerebellar ataxia type 6^3^).

Ocular albinism (OA) is a form of albinism in which the clinical features are constrained to the eye, whilst oculocutaneous albinism (OCA) encompasses a broader phenotypic range affecting the eyes, hair and skin^4^. The ocular manifestations of OCA and OA include infantile nystagmus syndrome (INS)^5^, foveal hypoplasia, abnormal crossing pattern at the optic chiasm and iris transillumination defects^6^ all of which can be subtle or incomplete^7^. Additionally, many of the ocular features seen in albinism can be seen in other disorders caused by mutations in genes that are not associated with melanin biosynthesis such as *PAX6* mutations, which can cause a variety of ocular phenotypes including nystagmus and foveal hypoplasia^8^. Similarly, Chediak-Higashi syndrome and Hermansky-Pudlak syndrome, caused by the *LYST* and *HPS* genes respectively, involve many OA and OCA phenotypic traits. Despite the significant systemic health implications of these forms of syndromic albinism, most patients never undergo genetic testing.

Genes involved in the melanin biosynthesis pathway are known to cause forms of both OA and OCA. Examples include *GPR143*, which is causal for OA1^9^, whilst *TYR*, *OCA2*, *TYRP1*, *SLC45A2*, *SLC24A5* and *C10orf11* are associated with OCA subtypes 1–4 and 6–7 respectively^4^. The OCA1 gene, *TYR*, is known to be associated with missing heritability^10,11^. Our group and others have previously reported a compound heterozygous tri-alleleic genotype in *TYR* which involves both rare (AF <5%) and common (AF 28–36%) functionally damaging variants which are likely to be on *trans* alleles^2,12,13^. The two common *TYR* variants p.S192Y and p.R402Q, have previously been shown to cause a 40% reduction in tyrosinase activity and protein misfolding, respectively^14,15^.

Pigmentary abnormalities in hair and skin may be apparent during phenotyping, although this is not always the case. Consequently, a range of potential diagnoses could be made in children with nystagmus, particularly between the ages of 4–6 months^10^.

As phenotyping becomes more precise and nuanced in children with nystagmus, it is possible that candidate gene lists can become more specific^16^. For example, an abnormal electroretinogram (ERG) can be the only indication that an underlying retinal dystrophy is the cause of the nystagmus. Hence, a retinal gene panel might be the most appropriate genetic testing option. It also allows potential candidate causal variant(s) to be interpreted with greater confidence. Previous studies of next-generation sequencing in INS patients have utilised large gene panels of up to 300 genes whilst identifying candidate causal variants in a recurrent, small subset of genes^17–19^, or in genes for conditions which would have been identified by basic phenotyping. This suggests that pre-selecting and interpreting variants in fewer genes for phenotyped patients, may provide the most efficient workflow and highest diagnostic yield in routine clinical practice.

## Aim

We describe the diagnostic yield of a clinically available 31 gene panel for nystagmus and albinism. We evaluate its clinical utility in both completely phenotyped and incompletely phenotyped patients in order to reflect the real-world limitations of phenotyping in the clinic and describe its use for patients presenting to specialist and non-specialist centres.

## Methods

### Ethics and consent

Consent was obtained in accordance with the Declaration of Helsinki and was approved by South West Hampshire Local Research Ethics Committee (LREC 028/04/t). University Hospital Southampton Research and development team approved the experiments and all experiments were performed in accordance with relevant guidelines and regulations. Informed consent was obtained from all subjects and, if subjects were under 18, from a parent and/or legal guardian.

### Patients

Eighty-one individuals (age range 0–18 yrs) were identified from a regional paediatric nystagmus clinic as having INS with or without clinical features suggesting albinism. All patients referred to a single, regional service were offered recruitment.

### Phenotyping

All patients underwent basic phenotyping as outlined in Norman *et al*.^2^. Briefly, this included history taking, orthoptic examination, age-appropriate visual acuity testing, anterior and posterior segment examinations and ERG. Visual evoked potential (VEP, using flash VEP for younger children and pattern onset for older patients) and optical coherence tomography (OCT) with the Leica OCT system or Spectralis OCT (Heidelberg Engineering) were performed in most patients. Eye movements were recorded in some subjects with the EYElink1000 + (SR research, Ottawa, Ontario, Canada) eye tracker. Patients with a diagnosis of a condition known to cause nystagmus (such as Down syndrome, congenital cataract or Aniridia) or where a specific diagnosis was strongly suspected (such as a cone disorder in a photophobic patient confirmed by ERG) or without an INS phenotype (such as Gaze Evoked Nystagmus, GEN due to cerebellar disease) and those who were born before 35/40 weeks gestation were excluded. For included patients, saliva samples (ORAGENE) were collected and DNA extracted using Oragene-DNA kit (OG-575) (DNA Genotek).

Probands were allocated into four phenotype subgroups; clinically IINS with complete phenotyping (group 1), clinically IINS with incomplete phenotyping (group 2), clinical phenotyping consistent with albinism with complete phenotyping (group 3), and clinical features suggestive of albinism with incomplete phenotyping (group 4) (Table 1).

**Table 1:** Selection criteria of the four, clinically relevant phenotype sub-groups.

Cohort sub-group number	Cohort sub-group	Predominant waveform direction	ERG	OCT	VEP-misrouting suggested	Iris trans illumination	Number of patients
1	Idiopathic nystagmus (with complete phenotyping)	Horizontal	Normal	Normal	No	No	18 (22.2%)
2	Idiopathic nystagmus (with incomplete phenotyping)	Horizontal or Equivocal	Normal or Equivocal	Normal or Untested or Equivocal	Normal or Untested or Equivocal	Normal or Untested or Equivocal	15 (18.5%)
3	Clinically consistent with Albinism (with complete phenotyping)	Horizontal or Multiplanar	Normal	Foveal hypoplasia	Yes	Yes	20 (24.7%)
4	Clinical features suggestive of Albinism (with incomplete phenotyping)*	Horizontal or Multiplanar	Normal or Equivocal	**Foveal hypoplasia** or Untested or Equivocal or Normal	**Yes** or Untested or Equivocal or Normal	**Yes** or Untested or Equivocal or Normal	28 (34.6%)

Equivocal results were those deemed insufficient to permit calling typically due to limited patient compliance or borderline responses for example crossed asymmetry identified on only a few runs of monocular VEP testing (as is commonly the case in patients with hypomorphic albinism). *Must include at least one of the features underlined.

### Next-generation sequencing

Eighty-one DNA samples were prepared across six batches using the Illumina TruSight One capture kit (Illumina 5200 Illumina Way San Diego, California USA) which targets 4811 clinically relevant genes. Next-generation sequencing was performed on the NextSeq. 500 platform.

### Gene panel

The UKGTN gene panel for ‘albinism and nystagmus’ (31 genes, accessed 29/05/2018) was used to prioritise genes for identification of candidate likely causal variants (See Supplementary Table S1).

### Bioinformatic pipeline

FastQ data were aligned to the hg38 human reference genome with BWA-MEM (0.7.12). GATK v3.7^20^ was used to call SNPs and short indels in a multisample VCF file. Annotation was performed using ANNOVAR v2015Dec^21^ to collate variant consequence, variant allele frequency (1000 Genomes Project, Exome Sequencing Project and Exome Aggregation Consortium) and pathogenicity scores with CADD^22^ and MaxEntScan^23^ for splice site variants. Further annotation was included from InterVar^24^ and Human Gene Mutation Database^25^. Coverage was determined using SAMtools v1.3.1^26^ and BEDtools v2.17.0^27^.

### Variant prioritisation

Variants were prioritised into two categories of ‘assumed pathogenic’ and ‘assumed likely pathogenic’. ‘Assumed pathogenic’ was defined as a variant which had a ‘pathogenic’ annotation in ClinVar, ‘pathogenic’ annotation by InterVar or ‘disease-causing mutation’ (DM) in HGMD. ‘Assumed likely pathogenic’ was defined as a variant which was: (1) not synonymous; (2) had an allele frequency ≤5% in 1000 Genomes Project (all populations), Exome Sequencing Project 6500 (all populations) *and* Exome Aggregation Consortium (all populations) and; (3) had *either* a CADD Phred ≥15^22^ or a MaxEntScan ≥|3|^28^. Variants which form part of a single likely causal genotype were identified as ‘likely causal’ variants whilst multiple possible causal genotypes were identified as ‘reportable likely causal’. Sanger sequencing was performed to verify ‘likely causal’ variants which were miscalled in >10% of individuals in the cohort. A Sanger sequencing primer pair was designed using ‘A Plasmid Editor (ApE)’ software spanning 19 bp (forward) and 20 bp (reverse) in length.

## Results

### Quality control

The mean read depth across all samples was 127X (See Supplementary Table S2) with 95.8% coverage at a depth of 20X or greater across the 31 gene panel.

### Assumed pathogenic variants

**Figure 1:**
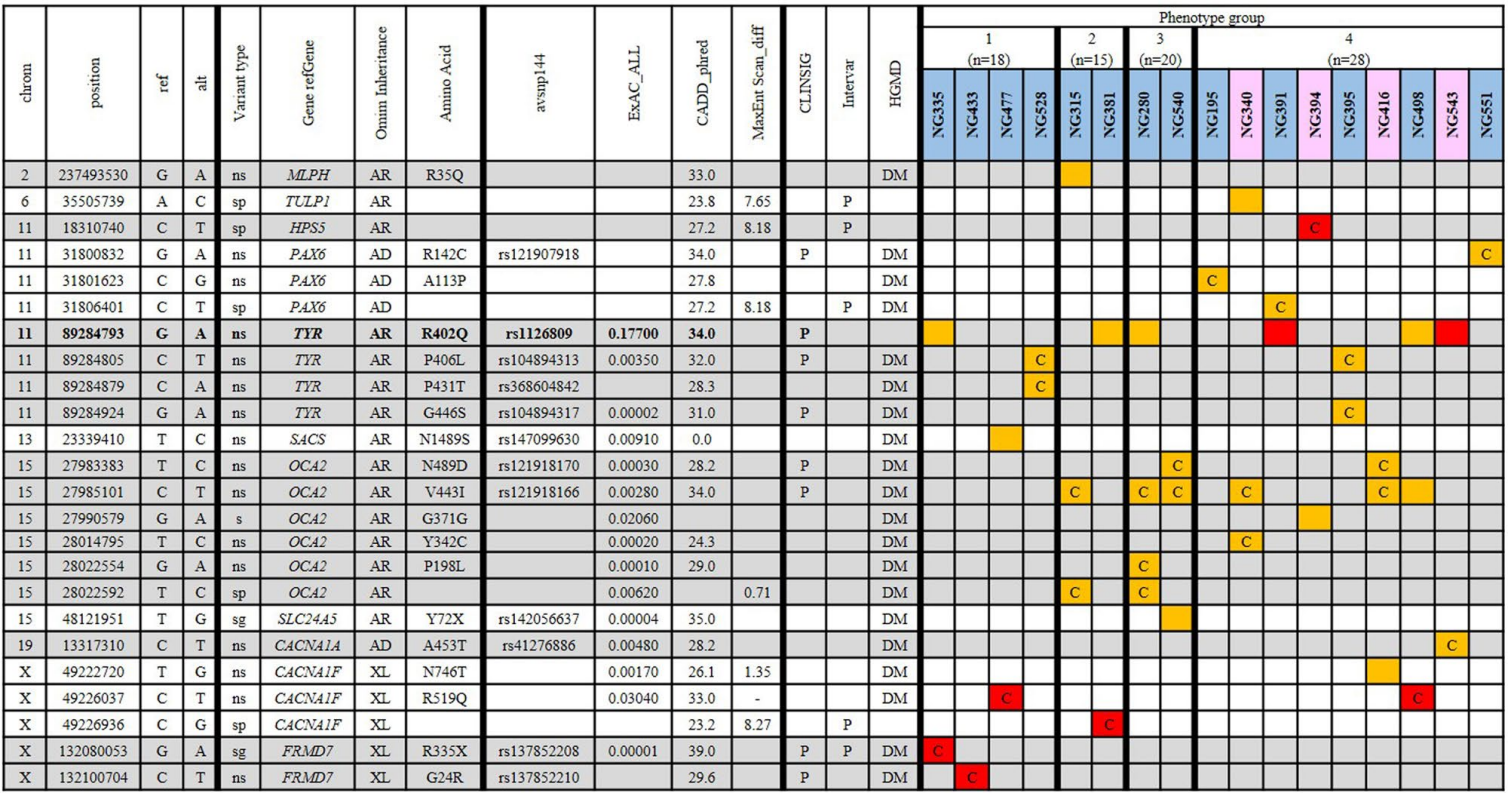
Seventeen of 81 patients with assumed pathogenic variants were determined to harbour likely causal genotypes. Samples, blue background indicates male whilst pink indicates female, orange cells indicate heterozygous variants and red cells indicate homozygous and hemizygous variants. Samples are ordered by phenotypic group, and cells with a ‘C’ denote assignment of a likely causal variant. The *TYR* variant, NM_000372:exon4:c.G1205A:p.R402Q (bold) would fail the MAF filter detailed above (17.7% AF in ExAC all populations) for putative variants but is highlighted here as it is listed as ‘pathogenic’ in ClinVar and is of relevance to subsequent work in this publication. Chrom, chromosome; Position, location of 5′ base of variant in hg38; Ref, reference allele; Alt, alternative allele; Variant type, consequence of the variant (s = synonymous, ns = nonsynonymous, sp = splicing, sg = stopgain); Gene.refGene, gene symbol; Omim Inheritance, inheritance as listed on OMIM for the gene in OCA/nystagmus; Amino acid, amio acid change; avsnp144, dbSNP144 rsID; ExAC ALL, Alternate allele frequency from ExAC database (all populations); CADD Phred, Combined Annotation Dependent Depletion score (Phred scale); MaxEntScan diff, the difference in score between MaxEntScan reference allele and alternative allele; ClinSig, clinical significance (clinvar) annotated ‘p’ if ‘pathogenic’; InterVar, annotated as ‘p’ if identified as ‘pathogenic’ by InterVar; HGMD 2016 CLASS, annotated as DM for disease-causing mutation; Variant category, 1 = assumed pathogenic, 2 = assumed likely pathogenic.

A total of 46 variants across the 81 participants met the criteria for assumed likely pathogenic genetic variants. For 17 patients (21.0% of the cohort), a total of 24 variants were considered to be likely causal variants (Fig. 1). Likely causal diagnoses were identified in 7 genes (*HPS5*, *PAX6*, *TYR*, *OCA2*, *CACNA1A*, *CACNA1F* and *FRMD7*) from the 31 gene panel. Twenty-two heterozygous variants that were initially labelled as assumed pathogenic or assumed likely pathogenic were found in genes known to cause recessive disorders in patients without a second identified putative variant.

### Assumed pathogenic and likely pathogenic variants

**Figure 2:**
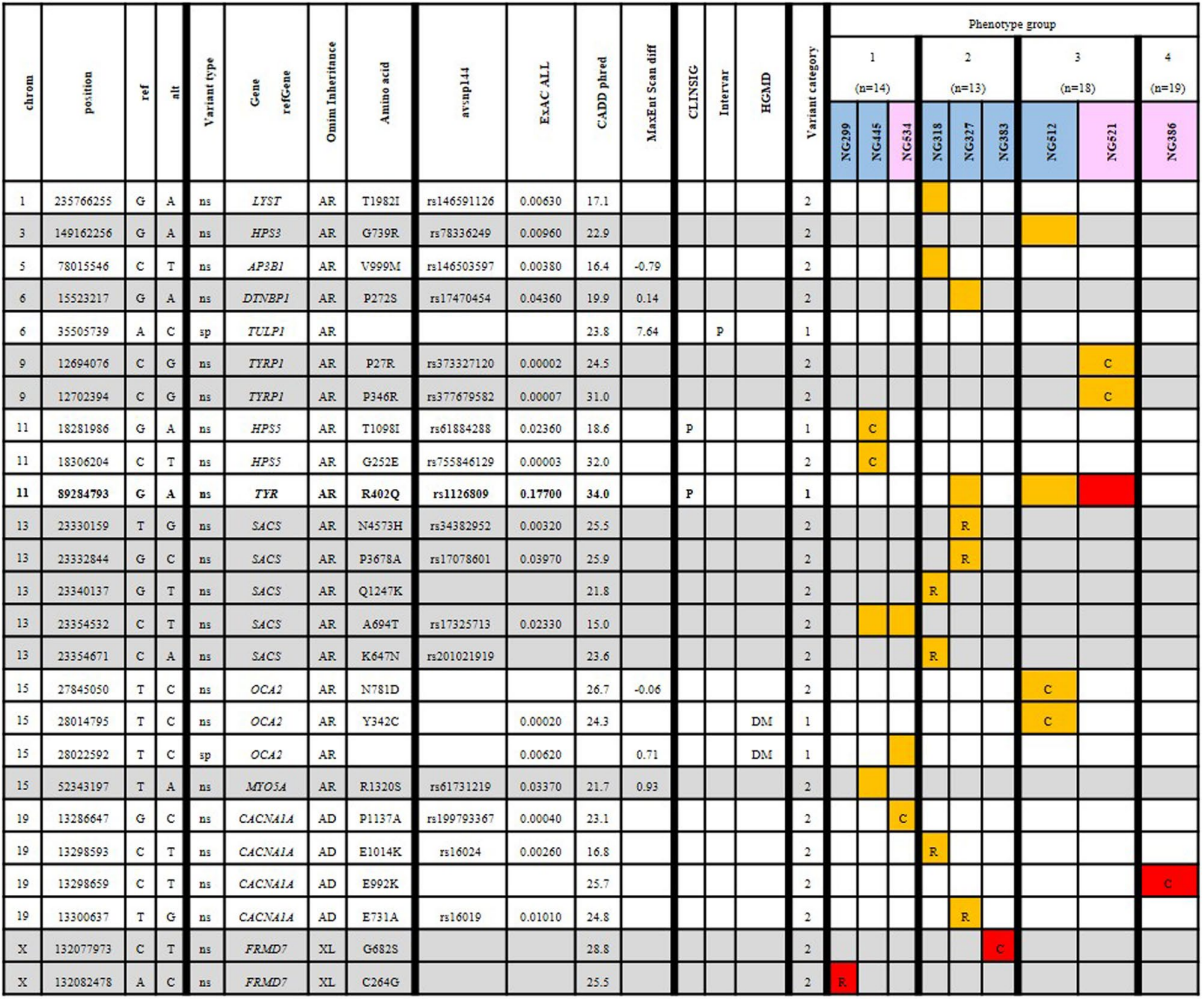
Nine patients with assumed likely pathogenic variants determined to be likely causal. For 64 patients investigated for likely causal genotypes by investigating variants which were assumed likely pathogenic or as a combination of assumed pathogenic and assumed likely pathogenic, 13 patients were determined to have likely causal genotypes. Samples, blue background indicates male whilst pink indicates female, orange cells indicate heterozygous variants and red indicates homozygous variants. Samples are ordered by phenotypic group, and cells with a ‘C’ denotes a likely causal variant,‘R’ denotes a reportable variant, ‘-’ denotes variant miscall. Chrom, chromosome; Position, location of 5′ base of variant in hg38; Ref, reference allele; Alt, alternative allele; Variant type, consequence of the variant (s = synonymous, ns = nonsynonymous, sp = splicing, sg = stopgain); Gene.refGene, gene symbol; Omim Inheritance, inheritance as listed on OMIM for the gene in OCA/nystagmus; Amino acid, amio acid change; avsnp144, dbSNP144 rsID; ExAC ALL, Alternate allele frequency from ExAC database (all populations); CADD Phred, Combined Annotation Dependent Depletion score (Phred scale); MaxEntScan diff, the difference in score between MaxEntScan reference allele and alternative allele; ClinSig, clinical significance (clinvar) annotated ‘p’ if ‘pathogenic’; InterVar, annotated as ‘p’ if identified as ‘pathogenic’ by InterVar; HGMD 2016 CLASS, annotated as DM for disease-causing mutation; Variant category, 1 = assumed pathogenic, 2 = assumed likely pathogenic.

For the remaining 64 patients without a likely causal genotype identified, assumed pathogenic variants together with assumed likely pathogenic variants (n = 89 unique variants) were interpreted for likely causality (Fig. 2).

Individuals with likely causal variants within the *PAX6* gene were attributed to two variants within the same codon, p.Q286. However, although these variants initially presented as variants of interest they had high failure rates (miscalls by GATK haplotype caller in 27.2% and 24.7% respectively of the total cohort). Subsequent verification with Sanger sequencing excluded these variants as false positives. For this reason, the two *PAX6* variants of the p.Q286 codon and an assumed likely pathogenic *HPS6* variant causing a p.W595G substitution (53.1% failure rate) were omitted from Fig. 2.

Nine of the 64 patients (11.1% of the total cohort) had likely causal diagnoses from assumed likely pathogenic variants with a cumulative total of 29 unique variants. Likely causal diagnoses were identified across 6 genes (*TYRP1*, *HPS5*, *SACS*, *OCA2*, *CACNA1A* and *FRMD7*) from the 31 gene panel. The remaining 58 heterozygous variants were found in genes known to cause recessive disorders in patients without a second identified putative variant.

### *TYR* tri-allelic genotypic cause of albinism

**Figure 3:**
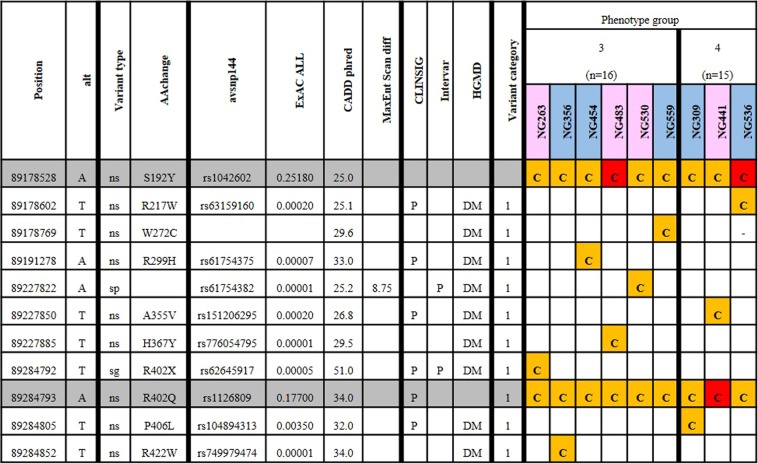
Nine samples were identified to have a likely causal tri-allelic genotype for albinism within *TYR*. For 55 patients which did not have likely causal genotypes with assumed pathogenic or assumed likely pathogenic variants, nine were identified to have tri-allelic causal genotypes in *TYR*. All *TYR* variants identified as assumed pathogenic or assumed likely pathogenic with the addition of S192Y and R402Q are listed. Position, location of 5′ base of variant in hg38; Alt, alternative allele; Variant type, consequence of the variant (s = synonymous, ns = nonsynonymous, sp = splicing, sg = stopgain); AAchange; avsnp144, dbSNP144 rsID; ExAC ALL, Alternate allele frequency from ExAC database (all populations); CADD Phred, Combined Annotation Dependent Depletion score (Phred scale); MaxEntScan diff, the difference in score between MaxEntScan reference allele and alternative allele; ClinSig, clinical significance (clinvar); InterVar, pathogenicity category according to InterVar interpretation; HGMD 2016 class, HGMD annotation for pathogenicity; Samples, orange indicates heterozygous variants, red indicates homozygous variants. Samples are ordered by phenotypic group, ‘c’ was used to indicate a likely causal variant, and grey highlights the common variants involved in the tri-allelic genotype outlined by Norman *et al*. (S192Y and R402Q).

The *TYR* variant, NM_000372.4:exon4:c.G1205A:p.R402Q satisfies our assumed pathogenic criteria as it is assigned as assumed pathogenic in ClinVar despite being very common (17.7% AF in ExAC all populations). This variant is thought to form a part of a tri-allelic phenotype^2,14,15^ with another common variant; NM_000372.4:exon1:c.C575A:p.S192Y and any other rare pathogenic *TYR* variant. Therefore, we consider it here as a unique case. Of the 55 remaining undiagnosed patients, nine were identified to have the tri-allelic genotype within *TYR* (Fig. 3). These nine patients originated from phenotype groups 3 (n = 6) and 4 (n = 3). Each patient had a minimum of the two common variants, NM_000372.4:exon1:c.C575A:p.S192Y (25.2% in all populations of ExAC) and NM_000372.4:exon4:c.G1205A:p.R402Q (17.7% in all populations of ExAC), and one rare assumed pathogenic variant which is deemed clinically sufficient to call as the molecular basis of albinism.

### Albinism patients with partially resolved genetic aetiology

In clinical practice it is common for gene testing to yield one well described pathogenic variant in an OCA gene but the absence of a second variant meaning that a molecular diagnosis cannot be made.

There were 46 patients without an assumed pathogenic, assumed likely pathogenic or *TYR* tri-allelic genotype identified. Forty-two patients across groups 1 (11), 2 (10), 3 (10) and 4 (15) without genetic diagnoses were subsequently investigated for single heterozygous assumed pathogenic or assumed likely pathogenic variants in an OCA/OA genes (*TYR*, *OCA2*, *TYRP1*, *SLC45A2*, *SLC24A5*, *C10orf11*, *GPR143*^2^). Strikingly, sixteen patients had a single assumed pathogenic or assumed likely pathogenic variant which are likely to contribute to an albino genotype (see Supplementary Fig. S1). This corresponds to 2/10 (20.0%), 7/10 (70.0%) and 7/15 patients (46.6%) for of the remaining unresolved cases for phenotype group 2, 3 and 4 respectively. No patients in group 1 had a single assumed pathogenic or assumed likely pathogenic variant in any albinism gene. This is insufficient for a clinical diagnosis but warrants further investigation as it strongly suggests other missing variants in albinism genes for these cases.

### Overview of diagnostic results

Table 2 summarises the number of samples harbouring likely causal variants. Overall, a clinically callable diagnostic yield of 48% was identified for the cohort as a whole. Phenotype groups 1 and 2 had similar diagnostic rates of 38% and 40% respectively. Groups 3 and 4 had a 50.0% and 57.1% diagnostic rate respectively. This shows that the additional phenotyping, beyond that of the baseline examinations prior to patient selection, did not significantly increase diagnostic yield.

**Table 2:** Summary table outlining the number of samples harbouring likely causal variants.

Group No.	Cohort sub-group	Samples	Samples with assumed pathogenic diagnostic variants	Samples with assumed likely pathogenic diagnostic variants	Samples with *TYR* tri-allelic genotype	% Samples with likely causal variants	Likely causal genes reported
1	Idiopathic nystagmus (with complete phenotyping)	18	4	3	0	38.9	*CACNA1A*, *CACNA1F*, *FRMD7*, *HPS5*, *TYR*
2	Idiopathic nystagmus (with incomplete phenotyping)	15	2	3	0	50.0	*CACNA1A*, *CACNA1F*, *FRMD7*, *OCA2*, *SACS*
3	Albinism/PAX6 disease diagnosis (with complete phenotyping)	20	2	2	6	50.0	*OCA2*, *TYR*, *TYRP1*
4	Albinism/PAX6 disease diagnosis (with incomplete phenotyping)	28	9	1	3	46.4	*CACNA1A*, *CACNA1F*, *HPS5*, *OCA2*, *PAX6*, *TYR*
		**81**	**17**	**9**	**9**	**43.2**	

A diagnostic rate is calculated and the likely causal genes are listed for each phenotype group.

## Discussion

In this study, we have utilised phenotyping methods which are currently employed in most large ophthalmology clinics worldwide. We recruited unselected, sequential patients in order to report on diagnostic yield using a UKGTN approved clinical panel based on the TruSight One ‘clinical exome’ panel which is being utilised in many centres as a cross-specialty, high throughput, sequencing platform. We report diagnostic yield for patients falling within the four most common clinical scenarios encountered in clinical practice; (1) complete phenotyped IINS, (2) likely IINS with incomplete phenotyping, (3) well phenotyped albinism and (4) likely albinism with incomplete phenotyping. We demonstrate a diagnostic rate across 81 patients of 43.2%, which is substantially higher than the majority of exome diagnostic analyses with the TruSight One capture^18^ and reflects, in part, the necessity for basic initial phenotyping in order to exclude common, clear clinical presentations and the utility of a subpanel of genes taken from a larger ‘clinical exome’ panel.

Six patients had assumed pathogenic variants in genes that would not have been previously directly implicated in causing the phenotype presentation of the patient according to the available clinical information. For example, NG315 from phenotype group 1 was found to have a likely disease-causing compound heterozygous genotype in the *OCA2* gene. These cases reflect the variable, often hypomorphic and overlapping phenotypes seen in children with nystagmus^4,29^, and support the argument that basic phenotyping alone prior to panel testing, may be the most efficient clinical diagnostic workflow rather than reducing the gene panel further by assuming that phenotyping has excluded or confirmed an albinism related phenotype.

The variant prioritisation category of assumed likely pathogenic identified some likely causal variants in genes for which the phenotype seen in our patients is unexpected. For example, NG381 (idiopathic nystagmus with incomplete phenotyping) was found to be homozygous for an assumed likely pathogenic splicing variant in the *CACNA1F* gene, which is known to cause Aland Island eye disease, cone-rod dystrophy and X-linked incomplete stationary night blindness (CSNB); all of which cause nystagmus and retinal dystrophy. It might be expected that such disorders would be identified by ERGs prior to recruitment to this study, however, this patient’s ERG result was initially reported as normal. Interestingly, a subsequent ERG performed at an older age for this patient identified the typical features of CSNB. This case and others may support an argument that genomic testing in the future may form an earlier part of the diagnostic workflow. More detailed phenotyping, which has been outlined here and by others^30^, might then be directed towards proving or disproving diagnoses suggested by putative likely causal variants, but it is clear that clinical evaluation and baseline phenotyping will still be required regardless of the yield from genetic testing.

The high number of cases identified here with likely albinism related phenotypes, but for whom only a single albinism gene pathogenic variant was identified, mirrors that seen in clinical practice. It strongly suggests that other, as yet unknown, variants in albinism genes are contributing or that multiple variants across the melanin biosynthesis pathway may combine to contribute to cause albinism phenotypes (epistasis). Some albinism genes such as the *C10orf11* gene are less well covered (70.5%) which may miss contributing variants for the albinism phenotype. The TruSight One chemistry could be backfilled to provide a more comprehensive and reliable capture of all target regions. Clearly, identification of these ‘missing variants’ and complex causal genotypes is likely to increase the diagnostic yield still further.

Nystagmus and albinism gene panels can vary from less than 20 to more than 300 genes^17,19,31^. Here we have used 31 genes taken as a subpanel from the commonly used TruSight One ‘clinical exome’ gene panel. The value of employing a large off the shelf target kit such as the TruSight One means that by revisiting the data already generated, the gene panel could be expanded retrospectively. The ‘TruSight One Expanded v3.0’ has also expanded on the original TruSight One capture to cover other known nystagmus-causing genes such as *SLC38A8*.

It is possible that a diagnostic report may initially include false positives, however, all identified variants which have been identified as ‘likely causal’ would be verified with Sanger sequencing before clinical reporting.

## Conclusions

In conclusion, the work presented here shows that for clinicians using a standard set of phenotyping criteria, the UKGTN approved 31 gene panel based on the TruSight One platform, can provide a clinically callable genetic diagnosis for 43.2% of children with INS regardless of more detailed phenotyping. This could significantly reduce the time and number of investigations that many children with nystagmus undergo and permit informed family counselling with regards to recurrence risk. This could also lead to more rapid access to tailored management, a current priority for health systems worldwide, for conditions such as spinocerebellar ataxia 6/episodic ataxia type 2 or Hermansky-Pudlak syndrome. For the future planning of diagnostic workflows and genetic testing strategies, it is important to note that the work here has shown similar diagnostic yield across all four patient groups. This suggests that clinical phenotyping (beyond the exclusion of retinal dystrophy, known underlying ophthalmic disease, prematurity or likely neurological cause) is not a key prerequisite for genetic testing, and that detailed phenotyping could be tailored subsequent to genetic testing in order to confirm or refute putative genetic diagnoses. It is also clear that diagnostic workflows for children with nystagmus should include differing genetic strategies according to the basic phenotyping results or availability of phenotyping modalities. For example, a first-line retinal gene panel for children with a retinal dystrophy identified by ERGs and a first line nystagmus/albinism gene panel for children with a clear albinism phenotype prior to ERG and VEP testing.

## Supplementary information

Supplementary Information

## Data Availability

Data generated or analysed during this study are included in this published article and its Supplementary Files.
