# Supplementary material for: A small gene sequencing panel realises a high diagnostic rate in patients with congenital nystagmus following basic phenotyping
Source: Sci Rep. 2019 Sep 13;9:13229. doi: 10.1038/s41598-019-49368-7 (PMC6744446; doi:10.1038/s41598-019-49368-7)
Supplement: Supplementary file 1 — Supplementary Information [file 41598_2019_49368_MOESM1_ESM.pdf]

Supplementary Information

A small gene sequencing panel realises a high diagnostic rate in patients with congenital nystagmus following basic phenotyping

Luke O'Gorman, Chelsea S. Norman, Luke Michaels, Tutte Newall, Andrew H. Crosby, Christopher Mattocks, Angela J. Cree, Andrew J. Lotery, Emma L. Baple, J. Arjuna Ratnayaka, Diana Baralle, Helena Lee, Daniel Osborne, Fatima Shawkat, Jane Gibson, Sarah Ennis[+], Jay E Self [+]*

[1]Human Development and Health, Faculty of Medicine, University of Southampton, MP808, Tremona Road, Southampton SO16 6YD, UK

[2]Clinical and Experimental Sciences, Faculty of Medicine, University of Southampton, MP806, Tremona Road, Southampton, SO16 6YD, UK

[3]Eye Unit, University Hospital Southampton NHS Foundation Trust, Tremona Road, Southampton, SO16 6YD, UK

[4]Institute of Biomedical and Clinical Science, University of Exeter Medical School, RILD Wellcome Wolfson Centre, Exeter, EX2 5DW, UK

[5]Wessex Investigational Science Hub, University Hospital Southampton, Tremona Road, Southampton SO16 6YD, UK

[6]Medical Research (Level 4), University of Exeter Medical School, RILD Wellcome Wolfson Centre, Royal Devon and Exeter NHS Foundation Trust, Barrack Road, Exeter, EX2 5DW, UK.

[7]Biological Sciences, Faculty of Natural and Environmental Sciences, University of Southampton, Southampton, SO17 1BJ, UK

[8]Human Genetics & Genomic Medicine, Faculty of Medicine, University of Southampton, MP 808, Tremona Road, Southampton SO16 6YD, UK

*J.E.Self@soton.ac.uk

[+]Joint last authorship

Table S1 ***UKGTN gene panel for 'Nystagnus and albinism'.*** *HGNC approved gene names for genes listed in the UKGTN gene panel for 'Nystagnus and albinism' with the associated OMIM inheritance pattern and phenotype.*

Symbol (HGNC)	Loci (HGNC)	Assumed inheritance pattern (OMIM)	Phenotype (OMIM)	Median Coding Sequence coverage at 20X depth of the n=81 cohort (RefSeq Curated)
AP3B1	5q14.1	AR	Hermansky-Pudlak syndrome 2	0.992
BLOC1S3	19q13.32	AR	Hermansky-Pudlak syndrome 8	0.943
BLOC1S6	15q21.1	AR	Hermansky-Pudlak syndrome 9	0.836
C10orf11	10q22.2-q22.3	AR	Albinism, oculocutaneous, type VII	0.705
CACNA1A	19p13.13	AD	Episodic ataxia, type 2	0.934
CACNA1F	Xp11.23	XL	Aland Island eye disease	0.985
CASK	Xp11.4	XLD	FG syndrome 4	0.980
DTNBP1	6p22.3	AR	Hermansky-Pudlak syndrome 7	0.993
FRMD7	Xq26.2	XL	Nystagmus 1, congenital, X-linked	0.961
GPR143	Xp22.2	XL	Ocular albinism, type I	0.844
HPS1	10q24.2	AR	Hermansky-Pudlak syndrome 1	0.929
HPS3	3q24	AR	Hermansky-Pudlak syndrome 3	0.994
HPS4	22q12.1	AR	Hermansky-Pudlak syndrome 4	0.977
HPS5	11p15.1	AR	Hermansky-Pudlak syndrome 5	0.975
HPS6	10q24.32	AR	Hermansky-Pudlak syndrome 6	1.000
LYST	1q42.3	AR	Chediak-Higashi syndrome	0.969
MANBA	4q24	AR	Mannosidosis, beta	0.991
MITF	3p13	AR	Tietz albinism-deafness syndrome	0.980
MLPH	2q37.3	AR	Griscelli syndrome, type 3	0.992
MYO5A	15q21.2	AR	Griscelli syndrome, type 1	0.987
OCA2	15q12-q13.1	AR	Albinism, oculocutaneous, type II	0.991
PAX6	11p13	AD	Foveal hypoplasia 1	0.945
RAB27A	15q21.3	AR	Griscelli syndrome, type 2	0.994
SACS	13q12.12	AR	Spastic ataxia, Charlevoix-Saguenay type	0.999
SETX	9q34.13	AR	Spinocerebellar ataxia, autosomal recessive 1	0.993
SLC24A5	15q21.1	AR	Albinism, oculocutaneous, type VI	0.995
SLC45A2	5p13.2	-	Albinism, oculocutaneous, type IV	0.939
TULP1	6p21.31	AR	Leber congenital amaurosis 15	0.981
TYR	11q14.3	AR	Albinism, oculocutaneous, type I	0.960
TYROBP	19q13.12	AR	Nasu-Hakola disease	0.988
TYRP1	9p23	AR	Albinism, oculocutaneous, type III	0.954

Table S2 Overall coverage and contamination metrics of 81 samples.

No. samples	Mean depth (X)	Mean coverage at 20x (%)	Min coverage at 20x (%)	Max coverage at 20x (%)	Mean hets (%)	Min hets (%)	Max hets (%)
81	127	95.8	90.6	0.987	65.2	57.4	72.2

Fig. S1 Patients with a partially resolved genetic aetiology in albinism genes. *Forty-six patients without an assumed pathogenic or assumed likely pathogenic likely causal genotype were identified across phenotype groups 1-4. These were analysed for single heterozygous assumed pathogenic or assumed likely pathogenic variants in at least one albinism gene (TYR, OCA2, TYRP1, SLC45A2, SLC24A5, C10orf11 and GPR143). Sixteen patients are shown to have an assumed pathogenic or assumed likely pathogenic variant in at least one albinism gene. TYR variant, NM_000372:exon4:c.G1205A:p.R402Q (bold) would fail the MAF filter detailed above (17.7% AF in ExAC all populations) for putative variants but is highlighted here as it is listed as 'pathogenic' in ClinVar. Samples, orange indicates heterozygous variants, red indicates homozygous variants. Samples are ordered by phenotypic group.*

																**Phenotype group**																
																**2 (n=10)**		**3 (n=10)**							**4 (n=15)**							
**chrom**	**position**	**ref**	**alt**	**Variant type**	**Gene refGene**	**Omim Inheritance**	**Amino acid**	**avsnp144**	**ExAC ALL**	**CADD phred**	**MaxEnt Scan diff**	**CLINSIG**	**Intervar**	**HGMD**	**Variant category**	**NG367**	**NG449**	**NG167**	**NG198***	**NG213**	**NG265**	**NG420**	**NG492**	**NG508**	**NG270**	**NG348**	**NG399**	**NG361**	**NG474**	**NG514**	**NG545**
5	33963893	C	T	nonsynonymous	*SLC45A2*	AR	C229Y	rs769099171	0.00001	25.3				DM	1																
9	12702394	C	G	nonsynonymous	*TYRP1*	AR	P346R	rs377679582	0.00007	31.0					2																
9	12704541			nonsynonymous	*TYRP1*	AR	T366M	rs199823942	0.00003	24.3					2																
9	12704558	G	A	nonsynonymous	*TYRP1*	AR	A372T			16.0					2																
10	76058747	G	T	nonsynonymous	*C10orf11*	AR	L160F	rs188514106	0.00190	18.5					2																
11	89178183	G	A	nonsynonymous	*TYR*	AR	R77Q	rs61753185	0.00010	33.0		P		DM	1																
11	89178482	G	T	nonsynonymous	*TYR*	AR	V177F	rs138487695	0.00001	26.7		P		DM	1																
**11**	**89284793**	**G**	**A**	**nonsynonymous**	***TYR***	**AR**	**R402Q**	**rs1126809**	**0.17700**	**34.0**		**P**			**1**																
11	89284805	C	T	nonsynonymous	*TYR*	AR	P406L	rs104894313	0.00350	32.0		P		DM	1																
15	27871156	T	A	nonsynonymous	*OCA2*	AR	M748L		0.00001	26.2	0.85	P			2																
15	27871170	G	A	nonsynonymous	*OCA2*	AR	P743L	rs121918167	0.00009	32.0		P		DM	1																
15	27926186	G	C	nonsynonymous	*OCA2*	AR	L674V		0.00030	27.3		P		DM	1																
15	27955156	A	G	splicing	*OCA2*	AR				23.5	7.75	P	P		1																
15	27983383	T	C	nonsynonymous	*OCA2*	AR	N489D	rs121918170	0.00030	28.2		P		DM	1																
15	27985101	C	T	nonsynonymous	*OCA2*	AR	V443I	rs121918166	0.00280	34.0		P		DM	1																
15	28016126	T	C	nonsynonymous	*OCA2*	AR	R290G		0.00002	22.6				DM	1																
